# Signatures of natural selection among lineages and habitats in *Oncorhynchus mykiss*

**DOI:** 10.1002/ece3.59

**Published:** 2012-01

**Authors:** Morten T Limborg, Scott M Blankenship, Sewall F Young, Fred M Utter, Lisa W Seeb, Mette H H Hansen, James E Seeb

**Affiliations:** 1 School of Aquatic and Fishery Sciences, University of WashingtonSeattle, Washington, USA; 2 National Institute of Aquatic Resources, Technical University of DenmarkVejlsøvej 39, Silkeborg, Denmark; 3 Washington Department of Fish and Wildlife600 Capitol Way N. Olympia, Washington, USA; 4 Department of Molecular Biology and Genetics, Faculty of Science and Technology, Aarhus UniversityDenmark

**Keywords:** Candidate genes, Interleukin, Local adaptation, MHC, Salmonid, Steelhead

## Abstract

Recent advances in molecular interrogation techniques now allow unprecedented genomic inference about the role of adaptive genetic divergence in wild populations. We used high-throughput genotyping to screen a genome-wide panel of 276 single nucleotide polymorphisms (SNPs) for the economically and culturally important salmonid *Oncorhynchus mykiss*. Samples included 805 individuals from 11 anadromous and resident populations from the northwestern United States and British Columbia, and represented two major lineages including paired populations of each life history within single drainages of each lineage. Overall patterns of variation affirmed clear distinctions between lineages and in most instances, isolation by distance within them. Evidence for divergent selection at eight candidate loci included significant landscape correlations, particularly with temperature. High diversity of two nonsynonymous mutations within the peptide-binding region of the major histocompatibility complex (MHC) class II (DAB) gene provided signatures of balancing selection. Weak signals for potential selection between sympatric resident and anadromous populations were revealed from genome scans and allele frequency comparisons. Our results suggest an important adaptive role for immune-related functions and present a large genomic resource for future studies

## Introduction

Inference of the structure and relatedness of natural populations exploded in the 1960s and 1970s with the development of molecular genetics and a deepening of our understanding of the genetic basis driving evolutionary change (e.g., Lewontin 1970). Our ability to resolve closely related populations evolved steadily through time with improvements in interrogation techniques (reviewed in Schlötterer 2004; Seeb et al. 2011a). Inference from single nucleotide polymorphisms (SNPs) during the last decade has sharpened our ability to observe differences among populations with addition of data from adaptively important loci (Anderson et al. 2005; Paschou et al. 2007; Helyar et al. 2011). The advances in studying functional genetic variation through genome scans (Storz 2005) have proven especially rewarding for studies aiming at linking phenotypic variations to a genotypic background in natural populations (Dalziel et al. 2009; Nielsen et al. 2009a).

**Figure 1:**
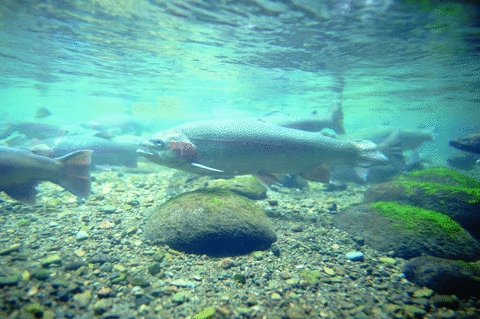
Wild rainbow trout (*Oncorhynchus mykiss*) in their natural environment (Photo by Finn Sivebæk).

**Figure 2:**
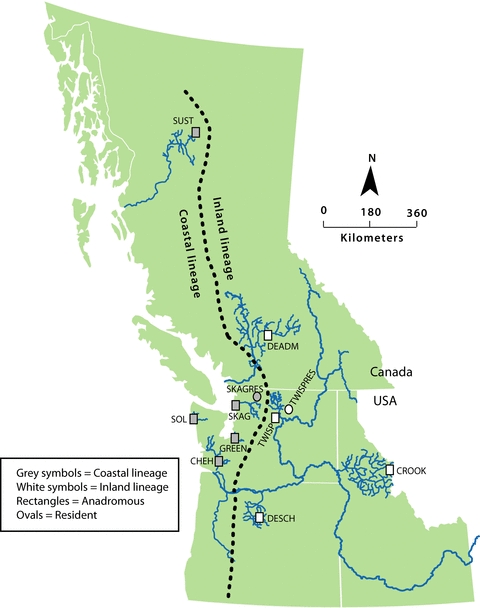
Map of sampling locations. An approximate projection of the current divide between the inland and coastal lineages is shown by a thick broken line (From Behnke 1992).

For decades, population genetics of Pacific salmonids has attracted substantial attention from both managers and researchers due to their economic importance as well as their complex biology where broadly diverse life histories have been described (reviewed in Utter 2004). A genetic basis for a range of different life histories, including oceanic migratory patterns, has been described over the years (see Quinn 2005 and references therein). The Pacific salmonid *Oncorhynchus mykiss* (Fig. 1) has been extensively studied reflecting its charisma in both recreational fisheries and aquaculture (Wishard et al. 1984; Jantz et al. 1990). *Oncorhynchus mykiss* has naturally colonized a range of habitats across the Beringial region from Kamchatka, Russia, in the west to Mexico in the southeastern part of its native distribution (MacCrimmon 1971). Wild populations have furthermore been successfully introduced throughout the world (MacCrimmon 1971) making it a model species for investigating local adaptation in the wild (e.g., Rubidge and Taylor 2004; Narum et al. 2008; Pearse et al. 2009; Narum et al. 2010b). In *O. mykiss,* two North American lineages likely predating the last glacial maximum (Allendorf and Utter 1979) included populations along a broad coastal region of the Pacific Northwest and distinct from those inland (also referred to as redband trout) primarily east of the Cascade Range in the Upper Columbia and Fraser Rivers (Fig. 2; Allendorf and Utter 1979; Utter et al. 1980; McCusker et al. 2000). These two clades are hereafter referred to as the coastal and inland lineages. Two distinct life-history forms of *O. mykiss* include anadromous steelhead, having extensive oceanic migrations, and purely freshwater resident rainbow trout. However, generally higher among-region than within-region genetic variation for sympatric steelhead and rainbow trout supports a polyphyletic nature of the assumingly derived resident life history (Docker and Heath 2003; Heath et al. 2008; Pearse et al. 2009). Most studies to date remain inconclusive regarding potential molecular adaptations and selective agents maintaining this life-history variation (e.g., Docker and Heath 2003; Heath et al. 2008).

Recent work using SNPs in nonmodel organisms has allowed increased knowledge about the role of functional genetic variation (Nielsen et al. 2009a). A large majority of SNPs are neutral and provide useful information about neutral evolution and demographic inference. However, SNPs residing within, or linked to, expressed genes such as those that encode for stress or immune responses, may encode alleles subject to natural selection and add insight into adaptive evolutionary processes (Morin et al. 2004; Bouck and Vision 2007). As key effectors of the adaptive immune system and displaying an unequaled level of polymorphism for coding genes, loci of the major histocompatibility complex (MHC) have received intense attention as candidates for genes under selection (e.g., reviews in Bernatchez and Landry 2003; Piertney and Oliver 2006). In teleost fishes alone, a 2008 review reported that the available sequence information included 3559 MHC class I and class II allelic variants from 137 species (Wegner 2008). Salmonids are particularly well suited for quantifying selective pressures, because of the minimalistic genetic architecture of their MHC loci: whereas other vertebrates possess multiple duplicated loci for both MHCI and MHCII, salmonids have just one locus with classical MHCI function (UBA), and one classical locus for each subunit of the MHCII (DAA and DAB) (Hansen et al. 1999; Landry and Bernatchez 2001). Nonclassical loci have been found for both MHCI (Dijkstra et al. 2006; Miller et al. 2006) and MHCII (Harstad et al. 2008), but these are highly divergent, characterized by low levels of polymorphism and are functionally different from the antigen-presenting classical loci.

In salmonid fishes, classical MHC loci have been investigated as being subject to balancing selection within and among natural populations (e.g., Miller et al. 2001; Aguilar et al. 2004) or divergent selection (Landry and Bernatchez 2001; Miller et al. 2001; Gomez–Uchida et al. 2011). The type of selection inferred for these genes has been shown to depend on the spatial scale considered with a tendency toward balancing selection acting at smaller regional scales (e.g., Miller et al. 2001). In contrast, patterns of divergent selection have often been inferred among populations at larger spatial scales and those inhabiting different environments (Bernatchez and Landry 2003). However, divergent selection has also been found at fine spatial scales between ecotypes of the same lake system (Gomez–Uchida et al. 2011; McGlauflin et al. 2011) indicating that factors such as habitat type or correlated variables are important drivers of selection at these genes. Thus, MHC markers show great potential for understanding and disentangling complex patterns of adaptive processes in natural populations inhabiting varying habitats such as salmonids in the Pacific Northwest.

The purpose of this study was to screen a new genome-wide SNP resource in *O. mykiss* for signatures of local adaptation over large parts of its native distribution. Defining populations as all genetically differentiated samples, we compared diverse spawning habitats representing different environmental regimes including two paired steelhead and rainbow trout populations within the same rivers. We will refer to steelhead and rainbow trout as anadromous and resident populations, respectively, in order to generalize our findings for other fish species exhibiting migratory life-history variation, including sockeye salmon (*O. nerka*) (Taylor et al. 1996) and brown trout (*Salmo trutta*)(Elliott 1994). Using a panel of 276 SNPs designed with the intent of spacing loci as widely as possible across the genome (including newly developed markers previously unscreened in natural populations), we find strong neutral structure between the two major lineages. We probed outlier loci for signatures of adaptation based on different habitats and life histories and found strong adaptive signatures for immune-related genes.

## Materials and Methods

### Sampling

We analyzed 823 individuals representing 15 collections (*n* = 24–95) of *O. mykiss* from nine rivers throughout the Pacific Northwest of North America (Table 1; Fig. 2). Our collections include sampling of sympatric anadromous and resident fish from the Twisp River and sampling of allopatric anadromous and resident populations from the Skagit River (see Table 1). Temporal replicates of populations from four locations were also analyzed (Table 1) to increase sample sizes and assure consistency in observed spatial structure (Waples 1990). For Twisp River collections, all resident fish were collected further upstream compared to sampling of sympatric anadromous fish. Residents were typically collected during July, and fish were targeted visually by size (>180 mm), robust body shape, coloration (visible parr marks, spotting, bold color), or evidence of spawning (wounds, scale loss, fin tears, expressing milt); however residents were collected from many areas that were known spawning locations of steelhead. In contrast, the resident collection from the North Fork Cascade River in the upper Skagit River system (SKAGRES) was expected to represent an upstream resident population physically isolated from downstream anadromous populations (no upstream gene flow) due to the existence of an approximately 30-m high waterfall.

**Table 1:** Sample information and summary statistics. Sample size (*n*), expected (*H_E_*) and observed heterozygosity (*H_O_*), allelic richness (*A_R_*), and percent polymorphic SNPs are given. Statistics are given for pooled samples for locations with temporal replicates

	Population name	ID	Year	Lineage	Life history	*n*	*H_E_*	*H_O_*	*A_R_*	Percent polymorphic SNPs
1	Sustut River	SUST96	1996	Coastal	Anadromous	50	0.20	0.20	1.62	70%
	Sustut River	SUST97	1997	Coastal	Anadromous	45				
2	Skagit River	SKAG	2007	Coastal	Anadromous	59	0.25	0.24	1.80	89%
3	Skagit River	SKAGRES	2009	Coastal	Resident	23	0.09	0.09	1.45	55%
4	Green River	GREEN	2007	Coastal	Anadromous	35	0.22	0.22	1.73	80%
5	Sol Duc River	SOL	2009	Coastal	Anadromous	94	0.23	0.23	1.76	92%
6	Chehalis River	CHEH	2007	Coastal	Anadromous	95	0.22	0.21	1.69	82%
7	Deschutes River	DESCH	1999	Inland	Anadromous	95	0.18	0.18	1.60	75%
8	Crooked Fork Creek	CROOK99	1999	Inland	Anadromous	48	0.18	0.18	1.59	82%
	Crooked Fork Creek	CROOK01	2001	Inland	Anadromous	47				
9	Twisp River	TWISP	2008	Inland	Anadromous	81	0.19	0.18	1.65	85%
10	Twisp River	TWISPRES07	2007	Inland	Resident	25	0.20	0.18	1.71	86%
	Twisp River	TWISPRES08	2008	Inland	Resident	13				
11	Deadman Creek	DEADM97	1997	Inland	Anadromous	76	0.19	0.18	1.57	64%
	Deadman Creek	DEADM99	1999	Inland	Anadromous	19				

### Molecular analyses and number of SNP markers

Genomic DNA was extracted from fresh fin or operculum tissue using QIAGEN DNeasy 96 tissue kits (Qiagen, Valencia, California, USA). PCR amplification and genotyping was performed in 96.96 Dynamic Arrays using the Fluidigm IFC thermal cyclers and BioMark instruments following the protocols of Seeb et al. (2009). All genotypes were scored automatically using the BioMark Genotyping Analysis software (Fluidigm, San Francisco, California, USA) and verified by two independent scorers. Any discrepancies were reassessed and either kept as a consensus or discarded. Furthermore, eight individuals from each 96 DNA sample plate (i.e., 9% of all samples) were genotyped twice for one-third of the SNPs on independent arrays to ensure reproducibility of results. We screened 276 SNPs (compiled from Aguilar and Garza 2008; Brunelli et al. 2008; Campbell et al. 2009; Sanchez et al. 2009; Stephens et al. 2009; Narum et al. 2010a; Abadia–Cardoso et al. 2011; Hansen et al. 2011 and unpublished sources listed in Table S1), of which 10 did not conform to Hardy–Weinberg equilibrium (HWE) or were suggested to be in linkage disequilibrium (LD), and these were excluded from further statistical analyses (see results, Appendix 1) leaving 266 informative SNPs. Another 21 were situated in six pairs and three triplets within the same coding gene, and are consequently very tightly linked (Appendix 2; Table S1). For subsequent statistical analyses relating to neutral population structure, 12 of these, together with eight suggested outliers (*P* < 0.01), were discarded in order to assume neutrality and independence among a set of 246 remaining markers (results, Appendix 1). Genome scans and landscape genetics analyses, which are based on individual marker information, were based on 266 SNPs including all 21 SNPs in known linkage groups (see below, Appendix 1).

### Temporal stability of allele frequency distributions

Pairwise *F*_ST_ estimates among the 15 collections were generated in Arlequin 3.5 (Excoffier and Lischer 2010) using 10,000 permutations with *P* values corrected for multiple tests using the sequential Bonferroni method (k = 105) (Rice 1989). These tests revealed lower estimates between all pairs of temporally replicated collections (*F*_ST_ = −0.002–0.011) compared to all spatial comparisons (*F*_ST_ = 0.013–0.375). Assuming a conservative α value of 0.001, all temporal comparisons remained nonsignificant while spatial comparisons were all significant. Temporal replicates were pooled to optimize sample sizes for a total of 11 populations in subsequent analyses.

### Conformance to HWE and nonrandom segregation of SNPs

Conformance to HWE was tested independently for each locus in each of the 11 populations using the MC algorithm implemented in Genepop 4.0 (Rousset 2008). *P* values were corrected using the sequential Bonferroni method (k = 3069) (Rice 1989). Linkage was only known for those nine groups of SNPs that were ascertained in single Sanger sequencing reads (Hansen et al. 2011; Table S1). Thus, we simply tested for nonrandom segregation of all pairs of loci within each population using Fisher's tests for gametic LD as implemented in Genepop 4.0 (Rousset 2008). Due to the high number of tests performed (i.e., 36,315 for each population), no correction for multiple tests was performed since this approach would be overly conservative and likely underestimate truly significant relationships. We followed a hierarchical approach with the following criteria for assessing LD among markers: (1) only SNPs with a minor allele frequency (MAF) > 0.10 were considered due to an expectedly high number of false positives associated with low levels of variation, (2) only locus pairs showing more than 50% significant tests (*P* < 0.05) with at least six performed tests among 11 populations, (3) for all locus pairs showing significant LD, SNPs potentially involved in multiple pairs were discarded.

### Summary statistics

Individual global *F*_ST_ values were estimated for each locus in Genepop 4.0 (Rousset 2008) as well as over all loci. Mean expected (*H_E_*) and observed (*H_O_*) heterozygosity were calculated for each locus and population using GenAlEx 6.4 (Peakall and Smouse 2006). Allelic richness (*A_R_*), a measure of the number of alleles corrected for minimum sample size, was calculated for all populations using FSTAT v2.9.4 (Goudet 1995). GenAlEx 6.4 was used to report the percentage of polymorphic loci in each population.

### Spatial population structure and diversity

We used 246 neutrally behaving and individually segregating SNPs to recalculate pairwise *F*_ST_ estimates among all 11 populations in Arlequin 3.5 (Excoffier and Lischer 2010) using 10,000 permutations. We then used pairwise *F*_ST_ values (all *P* < 0.001) to generate a multi dimensional scaling (MDS) plot in ViSta 5.6.3 (Young 1996) for visualizing neutral population structure.

### Signatures of selection

To detect genomic regions under selection, we used a total of 266 SNPs including 21 SNPs from known groups of tightly linked SNPs. Inclusion of known linked SNPs is expected to increase the chance of finding signatures of selection, as even closely linked SNPs may differ substantially in their level of differentiation (Gomez–Uchida et al. 2011). To test for potential related bias, analyses were repeated with a reduced dataset not including linked SNPs. First, we performed a global genome scan for nine populations (i.e., omitting the two resident populations) using the model by Excoffier et al. (2009) as implemented in Arlequin 3.5 (Excoffier and Lischer 2010). This approach simulates a neutral distribution of *F*_ST_ (or *F*_CT_) in relation to observed heterozygosity. Observed values by locus were then projected onto this distribution, and loci lying above or below the simulated 99% confidence threshold for neutral variation were considered as candidates for divergent or balancing selection, respectively. We applied the hierarchical test by grouping populations into two groups representing the coastal and inland lineages (Table 1). We assumed a model of 10 simulated groups with 100 demes and performed 100,000 simulations. To further understand the spatial pattern of potential selection for all outliers (*P* < 0.01) detected for both *F*_ST_ (i.e., among all populations) and *F*_CT_ (i.e., between lineages here), we plotted major allele frequencies over all populations. We also performed a genome scan over all anadromous populations using BayeScan 1.0 (Foll and Gaggiotti 2008). We ran 10 pilot runs of 5000 iterations with an additional burn-in of 50,000 iterations and a thinning interval of 50 followed by a final sample size of 10,000. Results from the two genome scan approaches were compared and dual outliers were considered as strong candidates for diversifying selection.

To investigate divergent selection between migratory life-history types, we performed individual genome scans for each of the two within-river anadromous and resident population pairs (i.e., SKAG and SKAGRES as well as TWISP and TWISPRES) using 10 simulated demes and 100,000 simulations in Arlequin 3.5. Again, we plotted major allele frequencies over all populations for all outliers above the 99% confidence levels. BayeScan 1.0 was not considered here, since it is expected to perform poorly with few samples (Foll and Gaggiotti 2008).

### Environmental effects on adaptive variation

We further tested for associations between landscape variables and allelic distributions for each SNP to reinforce evidence of natural selection acting on outlier loci (as opposed to false positives) (e.g., Fraser et al. 2011). Underlying correlations between allele frequencies and landscape parameters may occur by chance due to either isolation by distance or to more similar landscapes between neighbor populations. If not taken into account, such neutral background noise is expected to lead to an increased false-positive rate (Coop et al. 2010). We applied the Bayesian linear model implemented in the software Bayenv (Coop et al. 2010) to correct this. This method uses a covariance matrix based on neutral markers to filter out signals from neutral population structure while testing for significant relationships between landscape variables and locus-specific allele distributions. Results are given for 266 SNPs (as described above), and each landscape variable as a Bayes factor (BF). This BF reflects the ratio of the posterior support given to a model where the landscape variable has a significant effect on allele distributions over an alternative model where there is no effect on the SNP. First, we estimated a covariance matrix using the 246 independently and neutrally behaving SNPs (see above). Then, we tested for correlations between each of 266 SNPs and the following variables: (1) precipitation, (2) maximum temperature, (3) minimum temperature, (4) elevation, (5) latitude, and (6) longitude (Appendix 3). We used multiple independent Markov chain Monte Carlo (MCMC) runs with chain lengths of 100,000 iterations to ensure convergence of the model.

### Genetic variation at MHC genes

Some of the loci that we used were previously annotated and represent potential candidate genomic regions for selection (Table S1). In this study, we restrict our *a priori* focus to six newly developed markers residing within the classical MHC class I (Omy_UBA3a, Omy_UBA3b, and Omy_UBA2a) and the classical MHC class II (Omy_DAB-431, Omy_DABb, and Omy_DABc) genes (Hansen et al. 2011; Table S1). We observed intriguingly high diversity at two of the MHC class II SNPs (Omy_DAB-431, Omy_DABb) known to be nonsynonymous (Hansen et al. 2011). To test for balancing selection on this gene, we reconstructed most likely haplotypes from the three SNPs in known linkage within the MHC class II gene (Table S1) using the program PHASE V2.1 by Stephens et al. (2001). We then used the Ewens–Watterson homozygosity test as implemented in Arlequin 3.5 (Excoffier and Lischer 2010) to test for balancing selection on reconstructed haplotypes within populations and overall assuming an infinite allele mutation model and using 10,000 simulations. This test compares the expected HW homozygosity based on observed haplotype frequencies (here designated as Observed *F* value) with a simulated value (Expected *F* value) expected at mutation drift equilibrium for a gene with a similar number of alleles (Ewens 1972; Watterson 1978), and where balancing selection will lead to smaller observed than expected *F* values.

## Results

### Laboratory analyses and tests for HWE and LD

We excluded 18 individuals with missing data at more than 50% of the loci, which likely reflected poor DNA quality. Six of the 276 SNPs that we initially screened showed significant deviation from HWE in five or more populations after correcting for multiple tests and were excluded from further analyses (Appendix 1; Table S1). We observed seven pairs of loci showing significant LD (*P* < 0.05) in more than half of the performed tests leading to the exclusion of four loci, of which some were involved in multiple pairs, to avoid potential pseudoreplication by including markers in LD (Table S1). Further analyses were based on a final dataset of 805 individuals representing 11 populations (*n* = 23–95) and 266 SNPs (Appendix 1).

### Summary statistics

Over all populations, these 266 SNPs showed varying levels of differentiation with global locus-specific *F*_ST_ values ranging from 0.00 to 0.68. The frequency of polymorphic loci varied from 55 to 92% among populations (Table 1). We observe intermediate levels of genetic diversity ranging from 0.09 to 0.25 (*H_E_*), 0.09 to 0.24 (*H_O_*), and 1.45 to 1.80 (*A_R_*) with highly reduced levels in the SKAGRES population (Table 1).

### Spatial population structure and diversity

**Figure 3:**
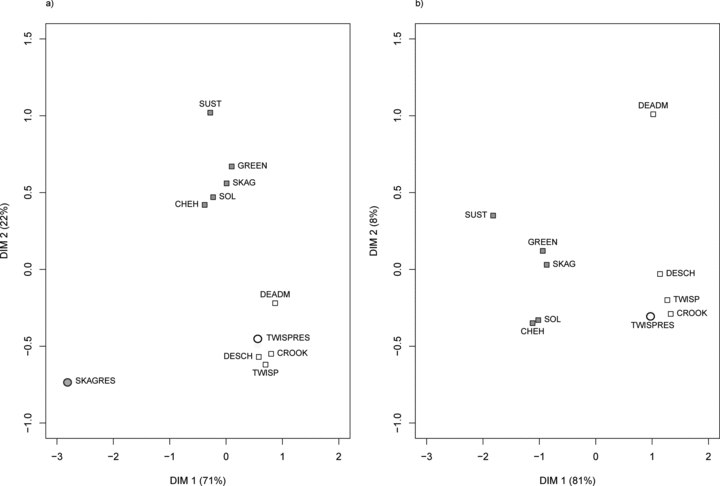
Multi dimensional scaling (MDS) plot showing: (a) spatial population structure for all populations including the two resident populations, and (b) a similar plot without the SKAGRES population. Population symbols follow Figure 2.

Spatial population structure inferred from significant pairwise *F*_ST_ values supported a pattern where most of the variation is likely caused by genetic drift and limited gene flow from historical isolation of the two lineages (Fig. 3). However, because most of the variation plotted for DIM 1 in the MDS plot was driven by the isolated SKAGRES population (Fig. 3A), omitting this population improved resolution for inferring spatial structure among remaining populations (Fig. 3B). The five coastal lineage populations cluster according to geography where the distant SUST population separates from a Puget Sound group (SKAG and GREEN) and a coastal Washington group (SOL and CHEH). Observed population structure within the inland lineage reflects contemporary geographic isolation with clear differentiation of the DEADM population (Canada) from remaining populations within the Columbia River drainage (Fig. 3B).

### Signatures of selection

**Figure 4:**
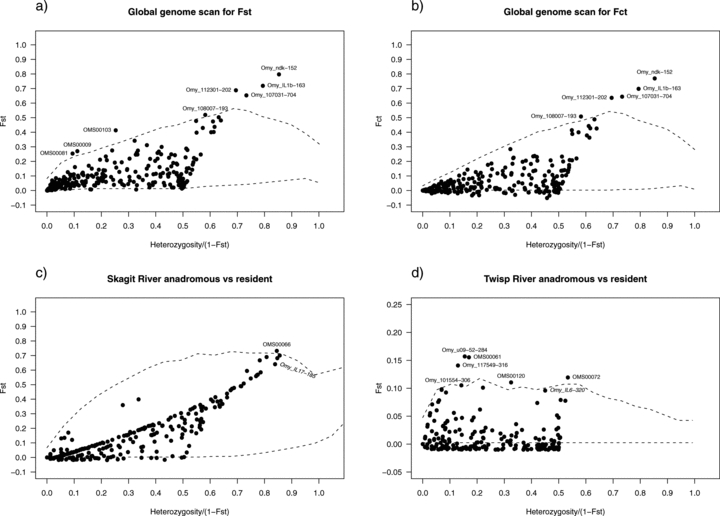
Outlier tests for identifying signatures of selection. (a) *F*_ST_-based global test assuming hierarchical structure by grouping all anadromous populations within each lineage into two major groups. (b) *F*_CT_-based global test assuming hierarchical structure as in (a). Local outlier tests for the Skagit River (c) and Twisp River (d) anadromous and resident population pairs are also shown. All outliers above the 99% confidence threshold are labeled including two interleukin genes at the 95% threshold (c and d) shown in italic. Plotted heterozygosity values are scaled by estimates of within population heterozygosity (*h*_0_) and locus specific *F*_ST_ as: (*H*_1_ = *h*_0_/[1 –*F*_ST_]) as described in Excoffier et al. (2009).

**Figure 5:**
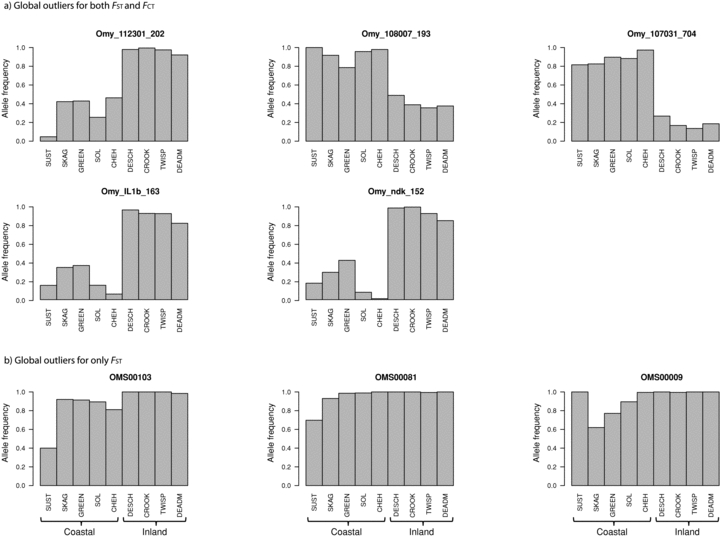
Frequency plots of major allele frequencies for loci detected as outliers (*P* < 0.01) in the global genome scan including nine anadromous populations. (a) Allele frequencies for five outliers detected at both the *F*_ST_ and *F*_CT_ level. (b) Allele frequencies for three outliers only detected at the *F*_ST_ level.

Candidates for balancing selection could not be distinguished from loci with observed *F*_ST_ values of zero, and we did not consider these further. The global genome scan assuming a hierarchical island model in Arlequin 3.5 revealed eight significant outliers for divergent selection (*P* < 0.01) at the *F*_ST_ level (Fig. 4A) of which five loci were also candidates at the *F*_CT_ level (Fig. 4B). This finding is more than twice as many as expected by chance alone (1% of 266 = 2.7). BayeScan detected four of the eight global outliers (*P* < 0.01) found with Arlequin as well as three new outliers (Table S1). These three outliers only detected by BayeScan were all characterized by very low minor allele frequencies (0.001–0.012), leaving them essentially uninformative. These were not considered, and we only interpret the eight candidates detected by Arlequin further. Functional roles could be inferred for two of the outliers, Omy_IL1b-163 and Omy_ndk-152, which reside within interleukin and nucleoside diphosphate kinase genes, respectively (Table S1; references therein). As expected, the five *F*_CT_ outliers reflect substantial differences between the two lineages (Fig. 5A), while all three outliers only detected at the *F*_ST_ level suggest a pattern of divergent selection within the coastal lineage as observed from deviating allele frequencies in the northern SUST compared to the other coastal lineage populations (Fig. 5B).

**Figure 6:**
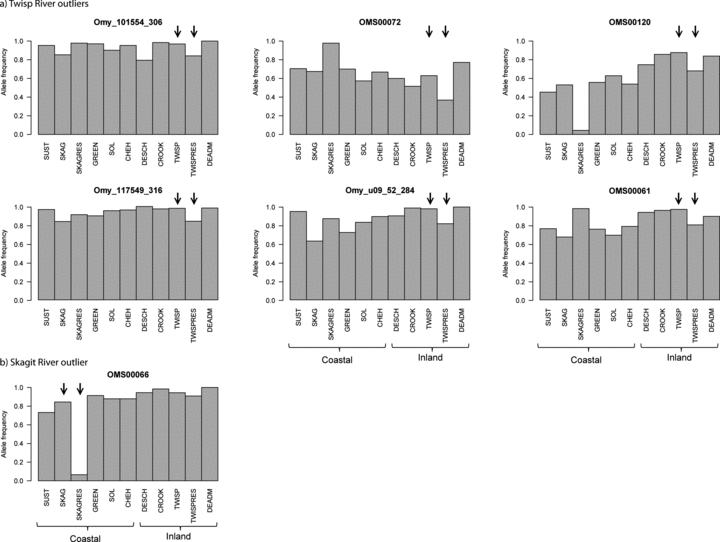
Frequency plots of major allele frequencies for loci detected as outliers (*P* < 0.01) between sympatric and resident population pairs. (a) Allele frequencies for six markers detected as outliers between the Twisp River populations. (b) Allele frequencies for one outlier detected between the populations within the Skagit River. Arrows denote populations included in the local genome scans.

Outliers under potential divergent selection (*P* < 0.01) were also detected in local genome scans for selection between within river populations exhibiting alternate life histories. We observe one outlier in the allopatric Skagit River and six in the sympatric Twisp River comparisons (Fig. 4C and 4D). Allele frequency plots for these local outliers all reveal a potential effect of anadromy. For example, allele frequencies for the anadromous Twisp River population are generally more similar to other inland anadromous populations compared to the sympatric resident population (Fig. 6A). For the outlier detected from the Skagit river populations, this pattern is even more pronounced (Fig. 6B). When also considering outliers at the 95% level, SNPs within interleukin genes (Omy_IL17–185 and Omy_IL6–320) are observed as outliers in the Skagit and Twisp River comparisons, respectively (Fig. 4C and 4D; Table S1). Another marker (Omy_97954–618) appears as an outlier for both locations at the 95% level suggestive of a consistent pattern of diversifying selection (Table S1).

### Environmental correlates

Bayesian inference for correlation between locus-specific allele distributions and landscape variables showed that 12 of 17 (71%) global candidates for divergent selection (*P* < 0.05; Table S1) significantly correlated with one or more variables (Table 2), contrasted with only 11 of 246 neutrally behaving loci (4%). When only considering “decisive” relationships (i.e., log10 (BF) > 2), only outlier loci correlated with any of the variables (Table 2). Particularly precipitation and temperature appear promising for explaining patterns of divergent selection at some of the candidate loci or linked genomic regions found here.

**Table 2:** Results from Bayesian inference of locus-specific landscape correlations. Gray cells denote a locus–parameter relationship with a log10 (BF) between 1.3 and 2.0, which can be interpreted as a *P*-value between 0.01 and 0.05. Black cells represent decisive relationships with log10 (BF) > 2.0 or equivalent *P*-values below 0.01. Here, global outliers include loci at the 5% significance level (see Table S1, Supporting information)

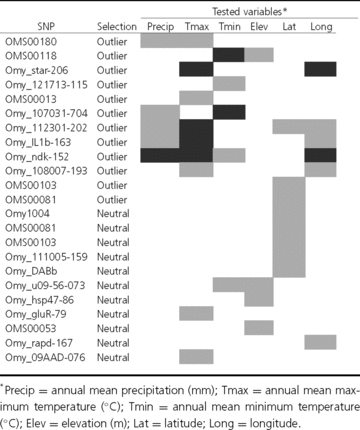

### Genetic variation at MHC genes

One SNP within the MHC class I gene (Omy_UBA2a) was discarded due to significant deviation from HWE (Table S1). Remaining MHC markers conformed to neutrality in genome scans coupled with low diversity in three of five markers (Appendix 4). However, the two nonsynonymous mutations residing within the MHC class II gene (Omy_DAB-431 and Omy_DABb) exhibit high levels of variation throughout most populations (Appendix 4). Reconstructed haplotypes based on three SNPs within the MHC class II gene revealed a significant deviation from neutrality toward balancing selection for two of 11 populations (Table 3). Furthermore, three populations had *P* values below 0.10 and all populations, except the Skagit River resident (SKAGRES), had smaller than expected *F* values pointing toward balancing selection (Table 3).

**Table 3:** For each population, number of reconstructed haplotypes, observed, and expected levels of homozygosity (*F* value) are given with results from the Ewens–Watterson homozygosity test for deviation from neutrality at an MHC class II gene (see text for more details). *P*-values below 0.05 are highlighted in bold, and *P*-values between 0.05 and 0.10 are shown in italic

Population	No. of haplotypes	Observed *F* value	Expected *F* value	*P*-value
CHEH	6	0.289	0.467	0.104
CROOK	6	0.295	0.468	0.113
DEADM	5	0.317	0.527	*0.076*
DESCH	5	0.255	0.530	**0.009**
GREEN	5	0.328	0.463	0.187
SKAG	5	0.313	0.500	*0.096*
SKAGRES	2	0.841	0.777	0.569
SOL	6	0.277	0.467	*0.079*
SUST	4	0.500	0.606	0.363
TWISP	6	0.248	0.457	**0.034**
TWISPRES	6	0.312	0.409	0.274
Mean	5.1	0.361	0.516	0.173
SD	1.2	0.173	0.101	0.168

## Discussion

We found highly significant spatial structure with increased levels of neutral differentiation between and within the two major lineages. This result is consistent with previous studies on *O. mykiss* from this region using allozymes and mtDNA (e.g., Allendorf and Utter 1979; McCusker et al. 2000). Applying multiple independent analytical steps (e.g., genome scans, landscape genomics, and raw allele frequency plots), the accumulated evidence supports local adaptation at several genomic regions including immune response genes.

### Spatial population structure and diversity

Most of the presumed neutral genetic variation that we observed can be explained by a model of historical vicariance. Distinct evolutionary lineages have seemingly accumulated genetic differentiation through genetic drift over glacial periods. Contemporary gene flow and drift appear less important at this large spatial scale but probably play a greater role at smaller regional scales among more recently diverged populations. This observation is supported by previous phylogenetic observations (Bagley and Gall 1998; McCusker et al. 2000) and early allozymes studies (Utter et al. 1980) showing similar patterns of strong differentiation between inland and coastal lineages compared to population structure within lineages. The location above a waterfall of the SKAGRES population likely explains its divergence (e.g., Fig. 3A) and low genetic diversity (Table 1) as a reflection of limited gene flow and low effective population size (*N_e_*) with consequently strong genetic drift. A similar scenario has been shown for another physically isolated population of resident *O. mykiss* (Pearse et al. 2009; Martínez et al. 2011). Omitting the SKAGRES population revealed further regional structure within the coastal lineage, suggesting more recent population histories potentially coupled with higher contemporary gene flow among populations within the Puget Sound and western Washington coastal regions, respectively (Fig. 3B). Despite the large spatial scale, these observations hold great promise for applying this SNP panel in future studies focusing on smaller geographic scales. Variation between the geographically distant populations from Sustut River and Deadman Creek in BC, Canada (Fig. 3B) is also apparent. The increased differentiation observed for the Canadian populations within respective lineages is likely a reflection of the substantial geographical separation coupled with longer divergence from the other populations. It is noteworthy how weak this latter signal is compared to that observed between lineages, suggesting a limited reflection of contemporary genetic drift compared to signals from historical separation.

Despite potential gene flow between the sympatric anadromous and resident Twisp River populations (see also Christie et al. 2011), our results suggest some level of neutral population structure (pairwise *F*_ST_ = 0.01; *P* < 0.001). Zimmerman and Reeves (2000) found evidence for reproductive isolation of sympatric steelhead and rainbow trout in the Deschutes River, Oregon, and explained this with variation in timing and location of spawning activities. A similar scenario with some level of spatio-temporal reproductive isolation of the two Twisp River populations would be in accordance with our observations.

### Signatures of spatially divergent selection

We detected eight candidates for directional selection among all populations (Fig. 4A). Five of these were in accordance with divergent selection between the coastal and inland lineages (Figs. 4B and 5A). Allele frequency plots (Fig. 5A) reveal high levels of information from the five *F*_CT_ outliers for distinguishing between the two lineages. Population history and dynamics of populations spawning within or in close proximity to the transition zone has been difficult to infer in previous studies applying fewer and assumingly neutral markers (Currens et al. 2009; Blankenship et al. 2011). Outliers observed in our study appear promising for future investigations of the nature (e.g., distinguishing neutral and adaptive genetic variation) and extent of this transition zone in *O. mykiss*.

Two outliers were known to reside within an interleukin gene (Omy_IL1b-163) and a nucleoside diphosphate kinase (Omy_ndk-152) gene (Table S1). A recent study also found Omy_ndk-152 and another SNP within an interleukin gene to be affected by selection in relation to anadromy at a much finer scale among populations within the Klickitat River (all derived from the inland lineage) draining into the Columbia River system (Narum et al. 2011). Our broad spatial representation of anadromous populations limits the ability to identify the geographic scale at which selection acts upon outlier loci. However, by applying a hierarchical island model and comparing outliers at the *F*_ST_ and *F*_CT_ levels, we can deduce whether divergent selective forces are likely to dominate within or between the two lineages (Fig. 5). A recent study by Meier et al. (2011) showed that both number and types of outlier loci for divergent selection varied substantially at different spatial scales in brown trout. This pattern demonstrates the need for denser sampling of populations if the goal is to increase the spatial resolution of inferred selective processes. The observed outliers might therefore be shaped by heterogeneous landscapes, or other selective agents, operating at smaller geographic scales within each lineage (e.g., Narum et al. 2008; Narum et al. 2010b). Indeed, our landscape genomics analysis suggested an important link between landscape variables and several loci (Table 2). Due to the inherent uncertainty of correlations between predefined variables such as used in this study, we refrain from concluding direct functional relationships for specific loci or landscape parameters (see also Bierne et al. 2011). Nevertheless, looking at overall trends two main findings can be inferred from this analysis. First, genetic variation associated with surrounding landscape variables was dominated by outlier loci suggesting a general pattern of local adaptation to specific environments by *O. mykiss*. Second, precipitation and temperature (or correlated factors), in particular, may play important roles in shaping adaptive genetic variation in *O. mykiss*. An effect of temperature would be in accordance with three regional (*F*_ST_) outliers following a latitudinal trend within the coastal lineage (Fig. 5B). A recent study by Wenger et al. (2011) also suggests an important role of temperature and flow regime (expected to be partly correlated with precipitation) in determining the distribution of suitable habitat for *O. mykiss,* adding support for crucial adaptive roles of these environmental parameters. However, these association-based findings remain indirect in nature, but direct links between variations in genotype, phenotype, and fitness remain very rare for any organism. Future studies obtaining much denser genomic coverage (see e.g., Hohenlohe et al. 2010) will allow a more direct chromosomal location of the gene(s) actually under selection. Alternatively, surveillance of fully controlled populations allows to track gene frequencies over time after being exposed to a new environment (e.g., Barrett et al. 2008). Thus, our results can be seen as hypothesis generating for future studies specifically investigating effects of certain landscape variables or candidate genes.

### Migratory life-history types

We identified one outlier potentially under divergent selection between Skagit River resident and anadromous populations (Fig. 4C). For the Twisp River resident and anadromous populations, we found six putative outliers for divergent selection (Fig. 4D). The one outlier observed between the two Skagit River populations is consistent with that expected by chance alone. Furthermore, true outliers can be difficult to distinguish from false positives in this comparison considering the high levels of observed neutral differentiation between these two populations (pairwise *F*_ST_ = 0.30, *P* < 0.001). However, the observation of six outlier loci (i.e., 2.3%) at the 99% confidence level between the Twisp populations, together with another marker showing signatures of selection in both locations, suggest ongoing selection between anadromous and resident life-history types. This overall result is consistent with recent studies identifying signatures of divergent selection between different migratory variants in *O. mykiss* (Martínez et al. 2011; Narum et al. 2011). All outlier loci show allelic patterns suggestive of distinctions between resident populations and the anadromous counterparts within the same lineages. For example, allele frequency plots reveal a consistent pattern of higher similarity among all inland anadromous populations than between the anadromous and resident Twisp River populations (Fig. 6A). These differences are small and at best weak indicators of ongoing selection between life histories. However, a mere effect of increased drift in an assumingly smaller and more isolated resident TWISPRES population cannot explain these observations since a general trend of increased variation was observed for this population (Table 1; Fig. 6A). Despite this generally inconclusive pattern, these outliers may potentially prove rewarding in future studies with a more targeted focus on studying selection between these life histories.

### Evidence of selection acting on immune response genes

The two known nonsynonymous mutations in the peptide-binding region of a MHC class II gene (Omy_DAB_431 and Omy_DABb) generally showed high levels of diversity in most populations with MAF ranging between 0.26 and 0.48 (Appendix 4). Although only two populations gave significant results in direct tests for balancing selection acting on reconstructed haplotypes of the MHC class II gene, a clear overall trend toward balancing selection was revealed (Table 3). Lack of more significant findings may be due to limited statistical power from the limited number of alleles (Table 3) when reconstructing haplotypes from only three segregating SNPs. However, since these mutations change amino acids in the crucial peptide-binding region of the MHC class II molecules, we would expect that lack of balancing selection would have led to elimination of otherwise assumingly deleterious mutations in just a few generations. While our results are only indicative of balancing selection within or among populations, many previous studies have detected patterns of balancing selection acting on MHC loci in other salmonid fishes (Landry and Bernatchez 2001; Miller et al. 2001), including *O. mykiss* (Aguilar and Garza 2006). Furthermore, a recent study by Martínez et al. (2011) detected divergent selection on a microsatellite locus linked to a MHC class II gene between steelhead and an upstream isolated resident population of *O. mykiss*. Although not discussed by the authors, a general trend of reduced genetic diversity was observed in the landlocked resident population; however, this population exhibited increased levels of diversity at the MHC-linked marker in accordance with balancing selection within the resident population. More convincing conclusions about balancing selection can be obtained from analyses based on sequencing larger fragments of genes covering multiple polymorphic sites. McCairns et al. (2011) followed this approach for a fragment of the peptide-binding region of a MHC class II gene in stickleback (*Gasterosteus aculeatus*) and found similar evidence for balancing selection. Sequence-based analyses are in general expected to be more powerful for detecting balancing selection compared to individual marker based outlier tests (Renaut et al. 2010; Brieuc and Naish 2011; Narum and Hess 2011).

We also found interleukin genes among outliers in all three genome scans (Fig. 4). A recent study by Narum et al. (2011) also found interesting patterns for these three loci. They found Omy_IL-320 to be a candidate locus for anadromy in *O. mykiss* populations from the Klickitat basin in the Columbia tributary, Washington. This result is in agreement with our observations at this locus showing a signature of divergent selection between the resident and anadromous populations in the Twisp River (Fig. 4D). Furthermore, we observed outlier patterns for two other interleukin markers Omy_IL1b-163 and OmyIL17–185 (Fig. 4B and 4C). These two markers were observed to correlate with one or more environmental variables in the study by Narum et al. (2011), indicative of adaptive roles. For example, Narum et al. (2011) found the Omy_IL1b-163 locus to correlate with temperature, and this finding is also supported here at a larger spatial scale (Table 2). Despite the different spatial scales, our results together with the study by Narum et al. (2011) add strong support for an important adaptive role of interleukin genes in *O. mykiss*. Temperature tolerance, or factors correlating with temperature such as parasite abundance and virulence (e.g., Marcogliese 2008), have also been shown to infer selection on immune genes in other fish and animals in general (e.g., Kurtz et al. 2004; Sommer 2005; McCairns et al. 2011).

In conclusion, we observed interesting patterns of adaptive variation at both interleukin genes (divergent selection) and a MHC class II gene (balancing selection). Here, the latter is represented by three SNPs hitherto unscreened in wild populations. These candidate genes will inevitably prove valuable in future studies of *O. mykiss* investigating the evolutionary role of immune response processes.

### The promise of applying functional genetic variation in conservation genomics

Genome scans including functional genetic variation have proven very promising for identifying (and understanding) adaptively important genes and traits in nonmodel organisms (e.g., Namroud et al. 2008; Nielsen et al.2009b; Glover et al. 2010), also see Vasemägi and Primmer (2005) and Storz (2005) for reviews. First, identification of intraspecific adaptive variation among populations is crucial for identifying focal intraspecific population units of high conservation value. Further, identification of highly discriminatory loci will greatly increase power for use in management related assignment tests (e.g., Freamo et al. 2011) or mixed-stock analyses (Freamo et al. 2011; Seeb et al. 2011b) of natural populations. Future studies identifying adaptive variation are thus expected to contribute toward development of more effective conservation plans at the intraspecific level of wild nonmodel organisms.

## Figures and Tables

**Appendix 1:**
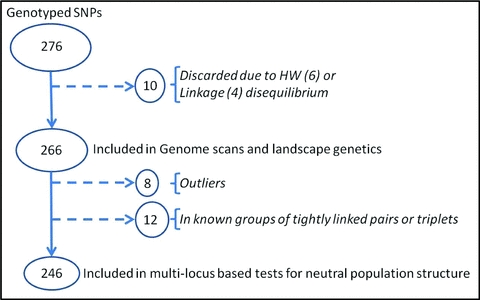
SNP exclusion pipeline. Ellipses isolate numbers of SNPs in the various categories. Solid arrows connect the stages of analysis, and broken arrows identify sets of SNPs that were excluded at each stage.

**Appendix 4:**
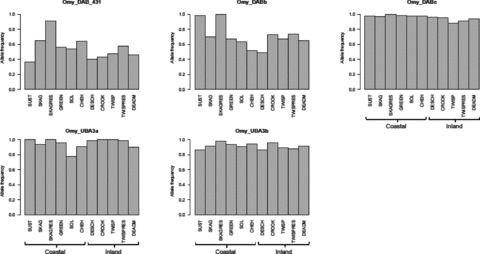
Frequency plots of major allele frequencies for five MHC-related SNPs. Omy_DAB_431 and Omy_DABb are nonsynonymous mutations in the peptide-binding region of a MHC class II gene, while Omy_DABc represents a synonymous mutation in the same gene. Omy_UBA3a and Omy_UBA3b show allele frequencies for two SNPs residing within a MHC class I gene.

## Appendix

**Appendix 2:** TaqMan assays for *O. mykiss* developed in the Washington Department of Fish and Wildlife (WDFW) Molecular Genetics Laboratory for genotyping steelhead. Gene targets were identified by BLASTX alignments in the Swiss-Prot database with E-values <1 and amino acid identities >25%

Assay name	Gene target	Design strand	Primers	Dye label	Probe sequences	Allele calls	Ascertainment set*
Omy_BAC-F5-284	Unknown	F	ACAACGCCAACAACTTTCTCTTG	VIC	CAGTAGGGCGGCAAG	C	1
		R	CCTCATTTACTGTAGGACCATGCA	FAM	ACAGTAGGACGGCAAG	T	
Omy_Il-1b_-028	Interleukin 1 beta_2	F	ACTGTCTGGCTAGAGCACATTG	VIC	CTGAGGCAACTTTTGT	T	2
		R	ATCTTCTACCACCGCACTGTTTTAA	FAM	TGAGGCAGCTTTTGT	C	
Omy_UT11_2a-018	Clock homolog	F	GCCTCCTGTGTATGAGTATGTTAAGTT	VIC	CTGAATAATGGTAAGTTTTC	T	2
		R	GCCAGCTACTTGTGTACTACATTCA	FAM	TGAATAATGGTACGTTTTC	G	
Omy_UT11_2a-114	Clock homolog	F	GCTGGCCTGTATTTGGTTTGTG	VIC	TGGTCGGTCTAAGTGC	C	2
		R	TTGGAGTTGAGTTAGGCACTATAGGA	FAM	TGGTCAGTCTAAGTGC	T	
Omy_UT11_2a-132	Clock homolog	F	GCTGGCCTGTATTTGGTTTGTG	VIC	AGGCAACAATGACTAATT	C	2
		R	CCATTTGGAGTTGAGTTAGGCACTA	FAM	AAGGCAACAATTACTAATT	A	
Omy_u07-79.166	Unknown	F	CCCGCTATATTATTTGATCACCCTTGA	VIC	ACTTGGGAATACCCCAGCC	G	1
		R	ATTTAAATCCATTTCTAAAAATAAGCAAACCTAACCA	FAM	CTTGGGAATAACCCAGCC	T	
Omy_u09-52.284	Unknown	F	TTTGTGTGTATTGTTGTGACTTG	VIC	ACTGCATTGTTGTAGCTAG	T	1
		R	TGATGTTATTGCAGGTCTAGCGAAA	FAM	CTGCATTGTTGTCGCTAG	G	
Omy_u09-53.469	Unknown	F	ACAGCCTGAGCGTTTGCA	VIC	TTGCAGCCCTTATTGTG	T	1
		R	GGAAACTGGGAGAGATCAAAGGA	FAM	TTGCAGCCCTTGTTGTG	C	
Omy_09-55-233	Unknown	F	CTCGTTTGATAGAAGAAACAAAGTGAAAGTG	VIC	AGCACTGACATCTGC	A	1
		R	CCAACATCTTTGGGCCTAAACAAGA	FAM	AGCACTGACATCCGC	G	
Omy_u09-56.073	Unknown	F	CCCACTACATCCTCATCCAAGGT	VIC	AGCGGCATTCTC	C	1
		R	CTCACTGCAAATCCAACTTCATCAT	FAM	CAGCGTCATTCTC	A	
Omy_u09-56.119	Unknown	F	CCAAGGTGGACCCACCAG	VIC	AGTGAGCTGAAACAGAGCA	T	1
		R	GCTGAGTTTATAGGTCAGTCATTATACATATTGA	FAM	TGAGCTGAAGCAGAGCA	C	
Omy_M09AAC-301	Polymerase (DNA directed),	F	CATTGATGGTTATTGTCATGCGTTTCA	VIC	CTGACAGATTTTGAAGTCT	T	1
	epsilon 2 (p59 subunit)	R	GCAGTAGAGATAGAAATTGAGCACACT	FAM	CTGACAGATTTTGACGTCT	G	
Omy_M09AAF-098	Exostosin like	F	CGGCGCCCGTCAGTA	VIC	CACAGGAGTAGTTAGAGTTA	T	1
		R	CAACCACCAGTCATGGCTCTA	FAM	CACAGGAGTAGTTCGAGTTA	G	
Omy_M09AAD-076	Family with sequence	F	ACTGTTACCACTCTCTCATCAACCT	VIC	CACCAACCACTGGTGAA	T	1
	similarity 203, member A	R	GGGTCCAGGAGGTTTTTAAACAACAT	FAM	CCAACCGCTGGTGAA	C	
Omy_09AAH-172	Rab-like protein 2A	F	GGCGTGAGCTTTGTGGTAGTA	VIC	AGGGATGCATACTCTG	T	1
		R	GCTGGACAGGAGCGGTTC	FAM	AGGGATGCATGCTCTG	C	
Omy_09AAI-092	Ubiquitin-like modifier	F	GGGCCTTGCTTTGCTTCTG	VIC	ATCTACTGAGTCTCTGCTGC	G	1
	activating enzyme 3	R	ACGGCTTAGATCCTCCTTGGA	FAM	CTACTGAGTGTCTGCTGC	C	

* Ascertainment set 1 included steelhead from Kamchatka, Russia (*n* = 3), Alaska, USA (*n* = 3), British Columbia, Canada (*n* = 3), Washington, USA (*n* = 180). Ascertainment set 2 included rainbow trout from Kamchatka, Russia (*n* = 10), Alaska, USA (*n* = 10), Washington, USA (*n* = 70), and cutthroat trout from Montana, USA (*n* = 10).

**Appendix 3:** Tested environmental variables and data sources

Environmental variable	SUST^1^	SKAG	GREEN	SOL	CHEH	DESCH	CROOK	TWISP	DEADM^1^
Precipitation (mm)^2^	965	873	1082	2501	1413	279	979	395	263
Maximum temperature (°C)^3^	21.0	22.7	24.6	20.0	24.8	32.2	28.0	29.5	26.2
Minimum temperature (°C) 4	–13.3	0.9	1.7	1.9	1.0	–3.5	–8.7	–9.6	–9.2
Elevation (m) 5	1352	9	22	9	28	397	1049	494	336
Latitude	56.58	48.44	47.29	47.91	46.80	44.82	46.51	48.37	50.74
Longitude	–126.45	–122.34	–122.17	–124.54	–123.17	–121.09	–114.68	–120.14	–120.92

Sources:

1 Precipitation and temperature data for the BC samples obtained from http://www.genetics.forestry.ubc.ca/cfcg/climate-models.html

2 http://www.prism.oregonstate.edu/products/matrix.phtml?vartype=ppt&view=maps.

3 http://www.prism.oregonstate.edu/products/matrix.phtml?vartype=tmax&view=maps.

4 http://www.prism.oregonstate.edu/products/matrix.phtml?vartype=tmin&view=maps.

5 Obtained from Google Earth using coordinates.

## Supporting Information

**Table S1.** Locus information and summary statistics.
